# Fraction of Inspired Oxygen With Low-Flow Versus High-Flow Devices: A Simulation Study

**DOI:** 10.7759/cureus.25122

**Published:** 2022-05-18

**Authors:** Yuki Kojima, Ryozo Sendo, Naoko Okayama, Junichiro Hamasaki

**Affiliations:** 1 Department of Anesthesiology, Asahi General Hospital, Asahi, JPN; 2 Department of Anesthesiology, Imakiire General Hospital, Kagoshima, JPN; 3 Department of Anesthesiology, Kagoshima City Hospital, Kagoshima, JPN

**Keywords:** fraction of inspired oxygen, respiratory system, respiration, oxygen, cannula

## Abstract

Purpose: The fraction of inspired oxygen while administering oxygen to patients must be measured as it represents the alveolar oxygen concentration, which is important from a respiratory physiology viewpoint. Therefore, the purpose of this study was to compare the fractions of inspired oxygen obtained through different oxygen delivery devices.

Methods: A simulation model of spontaneous respiration was used. The fractions of inspired oxygen obtained through low- and high-flow nasal cannulas and a simple oxygen mask were measured. The fraction of inspired air was measured every second for 30 s after 120 s of oxygen administration. This was measured three times under each condition.

Results: With a low-flow nasal cannula, airflow reduced both the intratracheal fraction of inspired oxygen and extraoral oxygen concentration, indicating that exhalatory respiration occurred during rebreathing and may be involved in increasing the intratracheal fraction of inspired oxygen.

Conclusion: Oxygen administration during expiratory flow may lead to an increased oxygen concentration in the anatomical dead space, which may be involved in the increase in the fraction of inspired oxygen. With a high-flow nasal cannula, a high fraction of inspired oxygen can be achieved even at a flow rate of 10 L/min. When determining the optimum amount of oxygen, it is necessary to set an appropriate flow rate for patients and specific conditions without being bound by the fraction of inspired oxygen values alone. It might be difficult to estimate the fraction of inspired oxygen while using a low-flow nasal cannula and simple oxygen mask in clinical situations.

## Introduction

Oxygen administration, performed in both acute and chronic phases of respiratory failure, is a common procedure in clinical medicine. The various modes of oxygen administration include an intubation tube, nasal cannula, oxygen mask, reservoir face mask, venturi mask, and high-flow nasal cannula (HFNC) [1-5]. The fraction of inspired oxygen (FiO_2_) represents the percentage of oxygen in the inspired air that is involved in alveolar gaseous exchange. The degree of oxygenation (P/F ratio) is the ratio of partial pressure of oxygen in arterial blood (PaO_2_) to FiO_2_. Although the diagnostic utility of the P/F ratio remains controversial, it is a widely used clinical indicator of oxygenation [6-8]. Therefore, it is clinically important to know the FiO_2 _value when administering oxygen to patients.

During intubation, FiO_2_ can be accurately measured using an oxygen monitor that includes a ventilator circuit, whereas when employing nasal cannulas and oxygen masks for oxygen delivery, only an “estimated value” of the FiO​​​​​​​_2_ can be measured based on the inspiratory time. This “estimated value” is the ratio of the amount of oxygen supplied to the tidal volume. However, it does not consider some factors from the viewpoint of respiratory physiology. Studies have shown that multiple factors affect FiO​​​​​​​_2_ measurements [2,3]. Although oxygen administration during expiratory flow may lead to an increased oxygen concentration in the anatomical dead space, such as the oral cavity, pharynx, and trachea, there are no reports on this topic in the current literature. Nevertheless, some clinicians argue that these factors are less important in practice, and the “estimated value” is sufficient to overcome clinical challenges.

In recent years, HFNC has attracted particular attention in emergency medicine and intensive medical care [9]. HFNC achieves a high FiO​​​​​​​_2_ and oxygen flow, and has two major advantages - pharyngeal dead space washout and reduction in nasopharyngeal resistance which should not be ignored when considering oxygen administration [10,11]. Additionally, it may be necessary to assume that the measured FiO​​​​​​​_2_ is the oxygen concentration within the respiratory tract or alveoli because the alveolar oxygen concentration during inspiration is important from the viewpoint of the P/F ratio.

Oxygen delivery methods other than intubation are commonly employed in routine clinical practice. Therefore, it is important to gather more evidence on FiO​​​​​​​_2_, as measured using these oxygen delivery devices, to prevent unnecessary over-administration of oxygen and to understand the respiratory status during oxygen administration in terms of safety. However, measuring FiO​​​​​​​_2_ in the trachea of the human body is difficult. Some researchers have attempted to simulate FiO​​​​​​​_2_ with a spontaneous respiration model [4,12,13]. Thus, in this study, we aimed to measure FiO​​​​​​​_2_ using a simulation model of spontaneous respiration.

## Materials and methods

Spontaneous breathing simulation model

**Figure 1:**
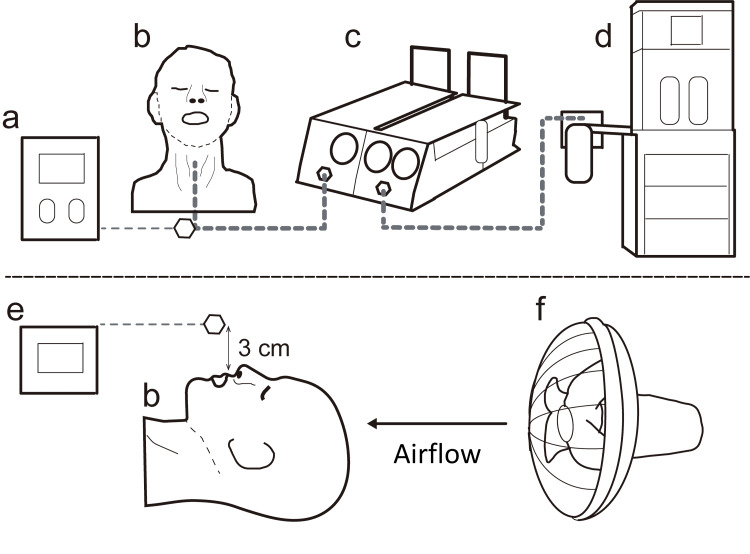
Experimental schema and actual experiment. (a) Oxygen monitor, (b) human manikin, (c) test lungs, (d) anesthesia apparatus, (e) oxygen monitor, and (f) electric fan. The ventilator settings were as follows: tidal volume, 500 mL; respiration rate, 10 breaths/min; and inspiratory-to-expiratory ratio (I:E ratio), 1:2 (respiration time = 1 s). The compliance setting of the test lungs was set to 0.5 for the experiment.

This is a simulation study and ethical approval was not required as this study did not involve human subjects. To simulate spontaneous breathing, we prepared a model of spontaneous breathing with reference to the model created by Hsu et al. (Figure 1) [12]. A ventilator from an anesthesia apparatus (Fabius Plus; Lübeck, Germany: Draeger, Inc.) and test lungs (Dual adult TTL; Grand Rapids, MI: Michigan Instruments, Inc.) were prepared to reproduce spontaneous respiration. The two devices were manually linked with a rigid metal strap. One bellow (the driving side) of the test lung was connected to a ventilator. The other bellow (the passive side) of the test lung was connected to the “oxygen administration model.” Once the ventilator delivered fresh gas to test the lung (the driving side), the bellow expanded, forcibly pulling the other bellow (passive side). This movement drew in gas through the trachea of the manikin, thus simulating spontaneous breathing.

Oxygen administration model

An oxygen monitor (MiniOx 3000; Pittsburgh, PA: Medical Services of America, Inc.) and human manikin (MW13; Kyoto, Japan: Kyoto Kagaku Co., Ltd.) were used for the oxygen administration model. Pure oxygen was administered at 1, 2, 3, 4, and 5 L/min, and each FiO_2_ was measured. For HFNC (MaxVenturi; Coleraine, Northern Ireland: Armstrong Medical), oxygen/air mixed gas was administered at 10, 15, 20, 25, 30, 35, 40, 45, 50, 55, and 60 L, and FiO_2_ was evaluated under each condition. For HFNC, experiments were conducted at oxygen concentrations of 45%, 60%, and 90%.

Evaluation of the Effect of Exhaled Respiration During Nasal Oxygen Administration

The extraoral oxygen concentration was measured (BSM-6301; Tokyo, Japan: Nihon Kohden Corp.) 3 cm above the maxillary incisors when oxygen was administered via a nasal cannula (Finefit; Osaka, Japan: Japan Medicalnext Co.) (Figure 1). Through the nasal cannula, air was blown from the head side of the manikin using an electric fan (HEF-33YR; Tokyo, Japan: Hitachi) to eliminate expiratory rebreathing, and FiO_2_ was measured after 2 min.

FiO_2_ Measurement

FiO_2_ was measured every second for 30 s, after 120 s of oxygen administration. After each measurement, the manikin and laboratory were ventilated. FiO_2_ was measured three times under each condition. The experiment began after calibrating each measuring instrument.

Estimated FiO_2_ Value (Nasal Cannula)

Traditionally, nasal cannula oxygen inhalation is assessed to allow for the measurement of FiO_2_. The calculation method used in this experiment changed depending on the content of spontaneous breathing (Table 1). The estimated value was calculated based on the respiratory conditions (tidal volume: 500 mL; respiration rate: 10 breaths/min; inspiratory-to-expiratory ratio {I:E ratio} = 1:2) set in the anesthesia device.

**Table 1:** Results of low-flow nasal cannula (LFNC). The “estimated value” is calculated at each oxygen flow rate. For LFNC oxygen administration, a nasal cannula was used. SD: standard deviation; FiO_2_: fraction of inspired oxygen.

	Intratracheal FiO_2_				Estimated value	Extraoral FiO​​​​​​​_2_			
	Without airflow		During airflow			Without airflow		During airflow	
	Average	SD	Average	SD		Average	SD	Average	SD
1 L	48.3	± 1.5	25.3	± 1.7	26.3	22.5	± 0.4	20.3	± 0.4
2 L	61.6	± 0.5	40.1	± 2.0	31.5	23.7	± 1.4	20.6	± 0.3
3 L	69.4	± 0.5	52.7	± 1.3	36.8	25.1	± 1.1	20.7	± 0.4
4 L	71.6	± 0.4	58.7	± 0.6	42.1	25.0	± 1.5	20.6	± 0.7
5 L	71.4	± 0.3	62.4	± 1.2	47.3	24.2	± 2.0	20.8	± 0.3

Statistical analysis

All analyses were performed using Origin software (Northampton, MA: OriginLab Corporation). The results are expressed as mean ± standard deviation (SD) for the number of tests (N) [12]. We have rounded up at the second decimal point in all results.

## Results

FiO_2_ measurement for low-flow nasal cannula

To calculate the “estimated value,” the oxygen taken into the lungs in a single inspiration was the amount of oxygen within the nasal cannula and the rest was outside air. Therefore, when the respiration time was 2 s, the oxygen administered via the nasal cannula in 2 s was 1000/30 mL. The oxygen dose taken from the outside air was 21% of the tidal volume (1000/30 mL). The final FiO_2_ was the amount of oxygen supplied to the tidal volume. Therefore, the “estimated value” of FiO_2_ could be calculated by dividing the total amount of oxygen consumed by the tidal volume.

**Figure 2:**
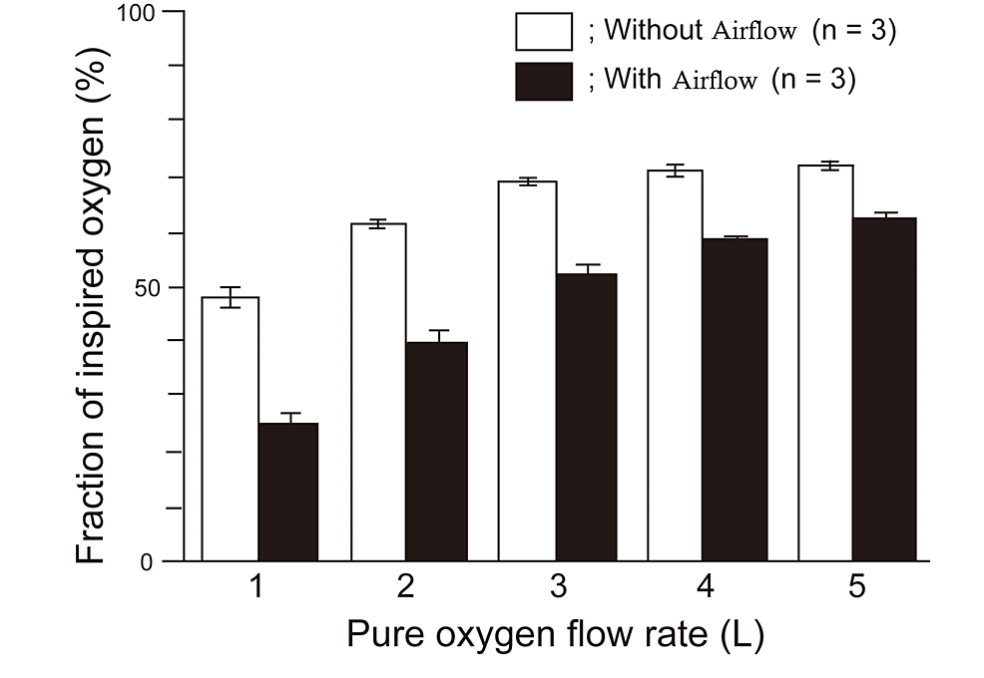
FiO2 with/without airflow in LFNC, the FiO2 differed with/without airflow. LFNC: low-flow nasal cannula; FiO_2_: fraction of inspired oxygen

Before every measurement, the calibration of the intratracheal oxygen monitor was 20.8% and that of the extraoral oxygen monitor was 21%. Table 1 shows the mean FiO_2_ values of the LFNC at each flow rate. These values were 1.5-1.9 times higher than the “estimated value” (Table 1). The extraoral oxygen concentration was higher than that of room air (21%). The mean values decreased before the introduction of airflow from an electric fan. These values were similar to the “estimated value.” During airflow while the extraoral oxygen concentration was approximated to that of the room air, the intratracheal FiO_2_ values were higher than the “estimated value” at more than 2 L/min. Regardless of the presence or absence of airflow, as the flow rate increased, the FiO_2 _difference decreased (Figure 2).

FiO_2_ measurement for a simple oxygen mask

Table 2 shows the mean FiO_2_ values of the simple oxygen mask (Ecolite oxygen mask; Osaka, Japan: Japan Medicalnext Co., Ltd.) at each oxygen concentration. These values increased as the oxygen concentration increased (Table 2). LFNC had a higher FiO​​​​​​​_2_ than did the simple oxygen mask at the same oxygen flow rate. The difference of FiO​​​​​​​_2_ was approximately 11-24% at 1-5 L/min.

**Table 2:** FiO2 measurement of the simple oxygen mask. For oxygen administration, a simple oxygen mask was used. SD: standard deviation; FiO_2_: fraction of inspired oxygen

	Intratracheal FiO_2_	
	Average	SD
1 L	30.9	± 0.9
2 L	37.7	± 0.7
3 L	47.0	± 1.9
4 L	54.9	± 1.2
5 L	60.6	± 1.2
6 L	68.6	± 0.5
7 L	71.6	± 0.6
8 L	74.7	± 0.9
9 L	76.8	± 0.6
10 L	79.6	± 0.6

FiO​​​​​​​_2_ measurement for high-flow nasal cannula

Table 3 shows the mean value of FiO​​​​​​​_2_ for HFNC at each flow rate and oxygen concentration. These values were close to the set oxygen concentrations, regardless of whether the flow rate was low or high (Table 3).

**Table 3:** FiO2 measurement with HFNC at each oxygen concentration. For HFNC, a high-flow system and flow driver were prepared. HFNC: high-flow nasal cannula; SD: standard deviation; FiO_2_: fraction of inspired oxygen.

	45%		60%		90%	
	Average	SD	Average	SD	Average	SD
10 L	41.1	± 0.8	54.1	± 1.5	88.6	± 1.4
15 L	42.6	± 0.4	56.7	± 0.4	89.5	± 0.6
20 L	42.5	± 0.2	56.9	± 1.2	89.6	± 1.0
25 L	42.3	± 0.2	57.1	± 1.8	89.6	± 0.7
30 L	42.1	± 0.1	57.2	± 2.1	89.3	± 1.0
35 L	42.1	± 0.1	57.2	± 1.8	89.3	± 1.1
40 L	41.8	± 0.3	57.3	± 1.3	89.2	± 0.6
45 L	41.8	± 0.3	57.1	± 1.4	88.6	± 0.4
50 L	41.7	± 0.2	57.1	± 1.3	88.2	± 0.2
55 L	41.5	± 0.2	57.2	± 1.3	88.0	± 0.5
60 L	41.4	± 0.3	57.2	± 1.4	87.7	± 0.3

## Discussion

While using the LFNC, the intratracheal FiO_2_ values were higher than the “estimated values” and extraoral FiO_2 _values were higher than those at room air. Flowing air was found to reduce both intratracheal FiO_2_ and extraoral FiO_2_. These results indicate that exhalatory respiration occurred during rebreathing with LFNC. Regardless of the presence or absence of airflow, as the flow rate increased, the FiO_2_ difference decreased. This result showed that another factor might be related to the increased intratracheal FiO_2_. In addition, they also indicated that oxygen administration increased the oxygen concentration in the anatomical dead space, which may be involved in the increase in FiO_2_ [2]. Conventional wisdom purports that LFNC does not cause expiratory rebreathing. It is speculated that this may have a significant effect on the difference between the measured value from the nasal cannula and the “estimated value.”

FiO​​​​​​​_2 _was lower for a simple mask than for a nasal cannula at low flow rates of 1-5 L/min, possibly because the oxygen concentration does not increase easily as the mask part becomes an anatomical dead space. In more than 5 L/min condition, flow oxygen minimized room air dilution, making FiO​​​​​​​_2_ steady [12]. At less than 5 L/min, a low point was noted in the FiO​​​​​​​_2 _value because of room air dilution and dead space rebreathing [12]. Indeed, the accuracy of the oxygen flowmeter can be highly variable. A MiniOx 3000 was used to monitor oxygen concentration; however, this device does not possess an adequate temporal resolution to measure variation in oxygen concentration over the breath (manufacturer quotes 20 s for a 90% response). This requires an oxygen monitor with much faster time response.

In actual clinical practice, the morphology of the nasal cavity, oral cavity, and pharynx differs among individuals, and the FiO​​​​​​​_2 _value may differ from the results obtained in this study. In addition, respiratory conditions vary among patients, and higher oxygen consumption will result in lower oxygen levels of exhalatory respiration. These conditions may result in a decrease in FiO​​​​​​​_2_ values. Therefore, it is difficult to estimate a reliable FiO​​​​​​​_2_ while using an LFNC and simple oxygen mask in actual clinical situations. However, this experiment suggests that the concept of anatomical dead space and rebreathing exhalatory respiration probably influences FiO​​​​​​​_2_. Given this finding, it is possible that FiO​​​​​​​_2_ may be sufficiently increased, even at low flow rates, depending on conditions rather than the “estimated values.”

According to the British Thoracic Society guidelines, clinicians should prescribe oxygen according to a target saturation range and monitor whether the patient’s condition is maintained within the target saturation range [14]. Although the “estimated values” of FiO​​​​​​​_2_ were very low in this study, it is possible to achieve an actual FiO​​​​​​​_2_ that is higher than the “estimated value” depending on the patient’s condition.

While using HFNC, the FiO​​​​​​​_2_ values were close to the set oxygen concentrations, regardless of whether the flow rate was low or high. The results of this study indicate that high FiO_2_ may have been achieved even at a flow rate of 10 L/min. Similar studies have suggested that FiO_2_ did not change between 10 and 30 L [12,15]. Reportedly, the high flow rate with HFNC eliminates the need to consider anatomical dead space [2,16]. It is possible that the anatomical dead space was washed out with an oxygen flow rate of more than 10 L/min. Dysart et al. posited that the principal mechanism of action of HFT may be flushing of the dead space of the nasopharyngeal cavity, thereby reducing overall dead space and increasing the fraction of minute ventilation that is alveolar ventilation [17].

A previous study of HFNC measured FiO​​​​​​​_2_ of the nasopharynx with a catheter, but the FiO​​​​​​​_2_ was lower than that in this experiment [15,18-20]. Ritchie et al. reported that the calculated FiO_2_ approached 0.60 as gas flow rates increased above 30 L/min during nose breathing [15]. In practice, a flow rate of 10-30 L/min or higher is required while using HFNC. Because of the characteristics of HFNC, the conditions in the nasal cavity have a considerable effect, and HFNC is often started at high flow rates. If respiratory conditions improve, it may also be necessary to reduce flow rates, as FiO​​​​​​​_2_ may be sufficient.

These results are based on simulations and do not imply that the findings of FiO​​​​​​​_2_ can be applied directly to actual patients. However, based on these results, in case of devices other than intubation or HFNC, it can be expected that the FiO​​​​​​​_2_ value will change significantly depending on the conditions. When oxygen is administered using an LFNC or simple oxygen mask in clinical situations, treatment is often evaluated by only the value of “oxygen saturation of the peripheral artery” (SpO_2_) using a pulse oximeter. In case the patient is anemic, it is suggested that the patient cannot be strictly managed without considering the values of SpO_2_, PaO_2_, and arterial oxygen content. In addition, Downs et al. and Beasley et al. have argued that unstable patients may actually be placed at risk with the precautionary use of high-concentration oxygen therapy [21-24]. During physiological deterioration, a patient who is administered high-concentration oxygen therapy would have a high pulse oximeter reading, which would mask the progressive decline in the P/F ratio, and therefore, the staff may not be alerted at the right time, leading to impending deterioration requiring mechanical support. It was previously believed that a high FiO_2_ is protective and provides patients a margin of safety; however, the theory is not applicable in a clinical situation [14].

Therefore, care should be taken even when oxygen is administered perioperatively or in the early stages of respiratory failure. The findings of the study suggest that an accurate measurement of FiO​​​​​​​_2_ cannot be obtained, except with intubation or HFNC. When using LFNC or a simple oxygen mask, it is necessary to ensure that oxygen is administered prophylactically to prevent mild respiratory failure. These devices may not be suitable when the respiratory status needs to be rigorously evaluated, especially in cases where FiO_2_ results are crucial. Even at low flow rates, FiO​​​​​​​_2_ increases with an increase in oxygen flow and may mask respiratory failure. Moreover, even when performing postoperative management with SpO_2_, it is desirable to have as low a flow rate as possible. This is necessary for the early detection of respiratory failure. A high oxygen flow increases the risk of early detection failure. It is desirable to determine the oxygen dose after confirming what vitals will be improved by administering oxygen. Based on the results of this study alone, it is not advisable to change the concept of oxygen administration. However, we believe that the novel ideas presented in this study should be considered in terms of the approach taken in clinical practice. In addition, when determining the amount of oxygen proposed by the guidelines, it is necessary to set an appropriate flow rate for patients without being bound by the FiO​​​​​​​_2_ value measured using the conventional inspiratory flow rate.

We suggest that the concept of FiO​​​​​​​_2_ should be reconsidered, considering the range of applications of oxygen therapy and the clinical setting, as FiO​​​​​​​_2_ is an indispensable parameter for guiding oxygen administration. However, there were few limitations to this study. If FiO​​​​​​​_2_ in the human trachea can be measured, more accurate values can be obtained. However, it is currently difficult to make such measurements without being invasive. Further research should be carried out with non-invasive measuring devices developed in the future.

## Conclusions

In this study, we measured intratracheal FiO_2_ with a simulation model of spontaneous respiration using an LFNC, a simple oxygen mask, and an HFNC. Oxygen administration during expiratory flow may lead to an increased oxygen concentration in the anatomical dead space, which may be involved in an increase in the fraction of inspired oxygen. With an HFNC, a high fraction of inspired oxygen can be achieved, even at a flow rate of 10 L/min. When determining the optimum amount of oxygen, it is necessary to set an appropriate flow rate for patients and specific conditions, without being bound by the fraction of inspired oxygen values alone. It might be challenging to estimate the fraction of inspired oxygen while using an LFNC and simple oxygen mask in clinical situations.

The study results suggest that exhalatory respiration is involved in the increase in intratracheal FiO_2_ with LFNC. When determining the amount of oxygen proposed by the guidelines, it is necessary to set an appropriate flow rate for patients without being bound by the FiO_2_ values, which were measured using the conventional inspiratory flow rate.
